# Migrating Staghorn Calculus Secondary to a Renocolic Fistula: A Case Report and Review of the Literature

**DOI:** 10.1155/criu/1014938

**Published:** 2025-02-26

**Authors:** Panagiota Fallon, Abhisekh Chatterjee, Nikolaos Chatzikrachtis, Dimitrios Sapountzis, Ivo Donkov, Samuel Bishara, Konstantinos Charitopoulos, Panagiotis Nikolinakos

**Affiliations:** ^1^Department of Urology, West Middlesex University Hospital, Chelsea and Westminster NHS Foundation Trust, London, UK; ^2^Department of Medicine, Faculty of Medicine, Imperial College London, London, UK; ^3^Department of Surgery, West Middlesex University Hospital, Chelsea and Westminster NHS Foundation Trust, London, UK

## Abstract

**Background:** Staghorn calculi are large renal stones which occupy most of the renal pelvis and are commonly associated with chronic or recurrent upper urinary tract infections (rUTIs). They often require more aggressive management, such as percutaneous nephrolithotomy (PCNL) or nephrectomy, although conservative management may be a safer option for select patients, particularly those with significant comorbidities. The presence of chronic stones or recurrent infections in the kidney increases the risk of complications, including fistula and abscess formation.

**Case Presentation:** A 68-year-old female presented to the emergency department with signs of urosepsis. Computed tomography (CT) imaging revealed a left-sided staghorn calculus with concurrent smaller renal calculi. Due to worsening kidney function during hospitalization, repeat imaging was done, which revealed a staghorn calculus in the rectum. The staghorn calculus migrated to the colon through a renocolic fistula, and the patient subsequently passed the large staghorn through the rectum spontaneously. Conservative management was pursued due to her high surgical risk. Several months after discharge, she represented with signs of infection, and a large left-sided psoas abscess was identified.

**Conclusion:** As the patient had severe comorbidities, our options to manage the staghorn calculi were very limited. She responded well to conservative management initially, but then was found to have another complication associated with the staghorn. It is acceptable to manage uncomplicated staghorn calculi conservatively in a small selection of patients, who are not good candidates for more invasive procedures, though in healthier and younger people, aggressive management is recommended to prevent further complications or deterioration. It is crucial to highlight the importance of early recognition and individualized treatment for renocolic fistulas, as timely intervention can significantly improve patient outcomes.

## 1. Introduction

Staghorn calculi are large, complex renal stones that form within the renal pelvis and are often associated with infections of the kidney. These stones account for 10%–15% of all kidney stones and are typically composed of struvite, a phosphate mineral produced by urease-producing organisms [1]. Due to the higher prevalence of urinary tract infections (UTIs) in women, staghorn calculi occur more frequently in females. These stones pose significant risks of morbidity and mortality, often requiring aggressive interventions such as percutaneous nephrolithotomy (PCNL) or even nephrectomy [2, 3]. However, conservative management may be safer in select patients with severe comorbidities or asymptomatic staghorn calculi [4].

Renocolic (or nephrocolic) fistulas are rare pathological entities and represent an abnormal connection between the renal parenchyma, or renal collecting system, and the colon. While iatrogenic factors, such as PCNL, or other abdominal surgeries, are the most common causes of renocolic fistula formation, these abnormal connections can also develop spontaneously. In the absence of external intervention, renal stones (including staghorn calculi) and recurrent infections can lead to local chronic inflammation and tissue ischemia, which subsequently causes tissue erosion and fistula formation [5]. This abnormal connection allows intestinal flora and pathogens to invade the normally sterile upper urinary tract, potentially leading to severe complications such as pyelonephritis [6], abscess formation [7], and obstructive uropathy [5].

We present the case of a 68-year-old woman with a staghorn calculus that passed through a renocolic fistula and was found in the rectum.

## 2. Case Report

### 2.1. Clinical Presentation

A 68-year-old-woman presented to the emergency department with a 2-day history of acute confusion and behavioural changes (delirium), against the background of known severe cerebral palsy. She was known to have recurrent UTIs well-managed at home with oral antibiotics. The rest of her past medical history included bilateral staghorn calculi, which had been previously treated by PCNL. On presentation, her blood results showed a marked increase in C-reactive protein (CRP = 260.9 mg/L), a decline in renal function (estimated glomerular filtration rate (eGFR) = 38 mL/min/1.73m^2^, urea = 11.7 mmol/L, and creatinine = 124), and microcytic hypochromic anemia with thrombocytopenia. The blood cultures that were taken returned as negative, and the urine cultures revealed mixed growth.

### 2.2. Diagnostic Approach

**Figure 1:**
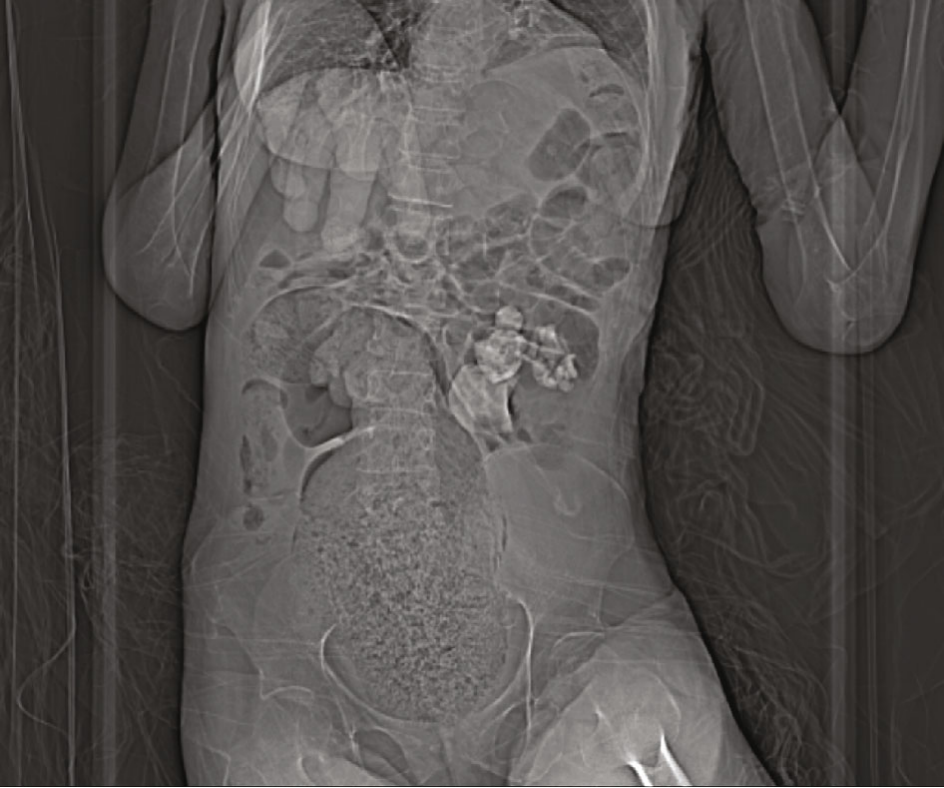
Initial CT scan scout film, showing the staghorn calculus located in the left kidney.

**Figure 2:**
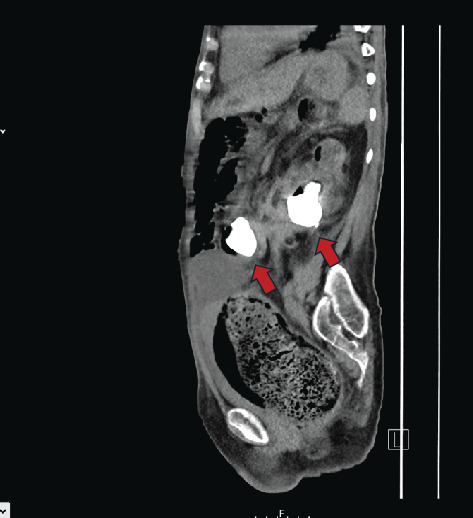
Initial CT scan, demonstrating the staghorn calculus within the renocolic fistula (left arrow) alongside the staghorn inside the left kidney (right arrow).

**Figure 3:**
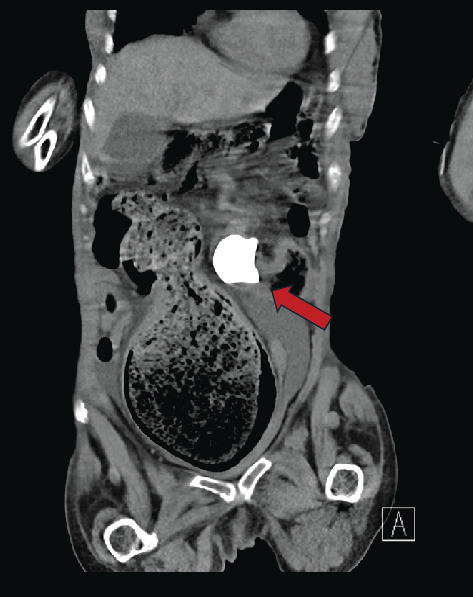
Coronal plane of the initial CT scan, showing the staghorn calculus within the renocolic fistula, measuring 4.5 cm. The migrated staghorn is seen adjacent to the distended large bowel and not in the anatomical position of the right renal pelvis.

**Figure 4:**
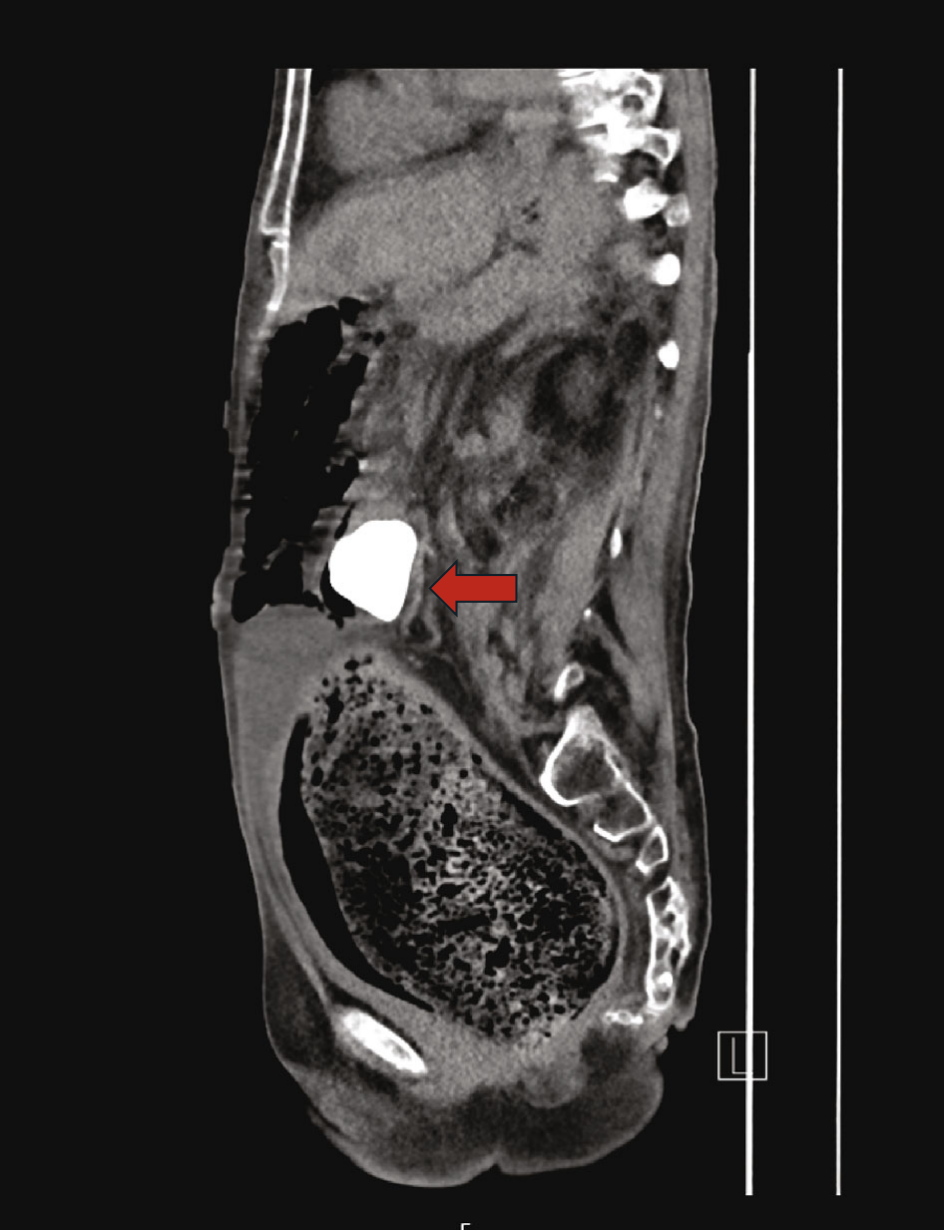
Sagittal plane of the initial CT scan, displaying the staghorn calculus within the renocolic fistula, measuring 3.9 cm.

The initial diagnosis included a UTI, acute kidney injury (AKI), and microcytic anemia with thrombocytopenia, all in the context of urosepsis. As she was severely constipated, an abdominal X-ray was ordered, which first revealed left radiopaque renal calculi. A subsequent computed tomography (CT) KUB (kidneys−ureters−bladder) scan identified large left renal calculi, including a left staghorn calculus measuring 4.5 cm. The presence of a second displaced left-sided staghorn (measuring 4 cm) within a renocolic fistula was initially missed. The migrated staghorn was described as “calcification arising from the large bowel.” The presence of a renocolic fistula was suggested as there were pockets of air seen inside the renal pelvis (Figures 1, 2, 3, and 4).

**Figure 5:**
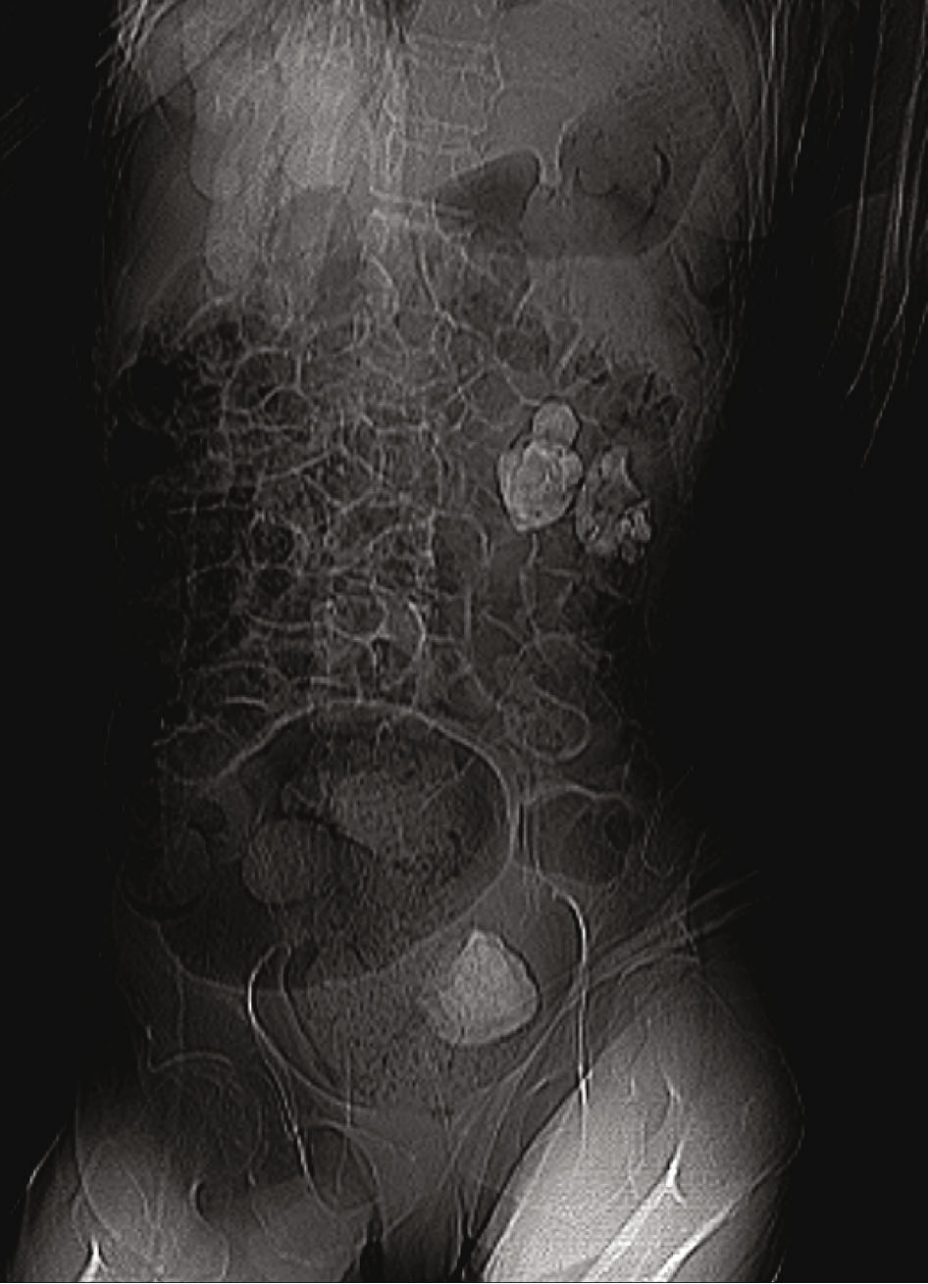
Scout film derived from the repeat CT KUB scan, revealing a staghorn calculus within the rectum, along with several large calculi inside the left kidney.

**Figure 6:**
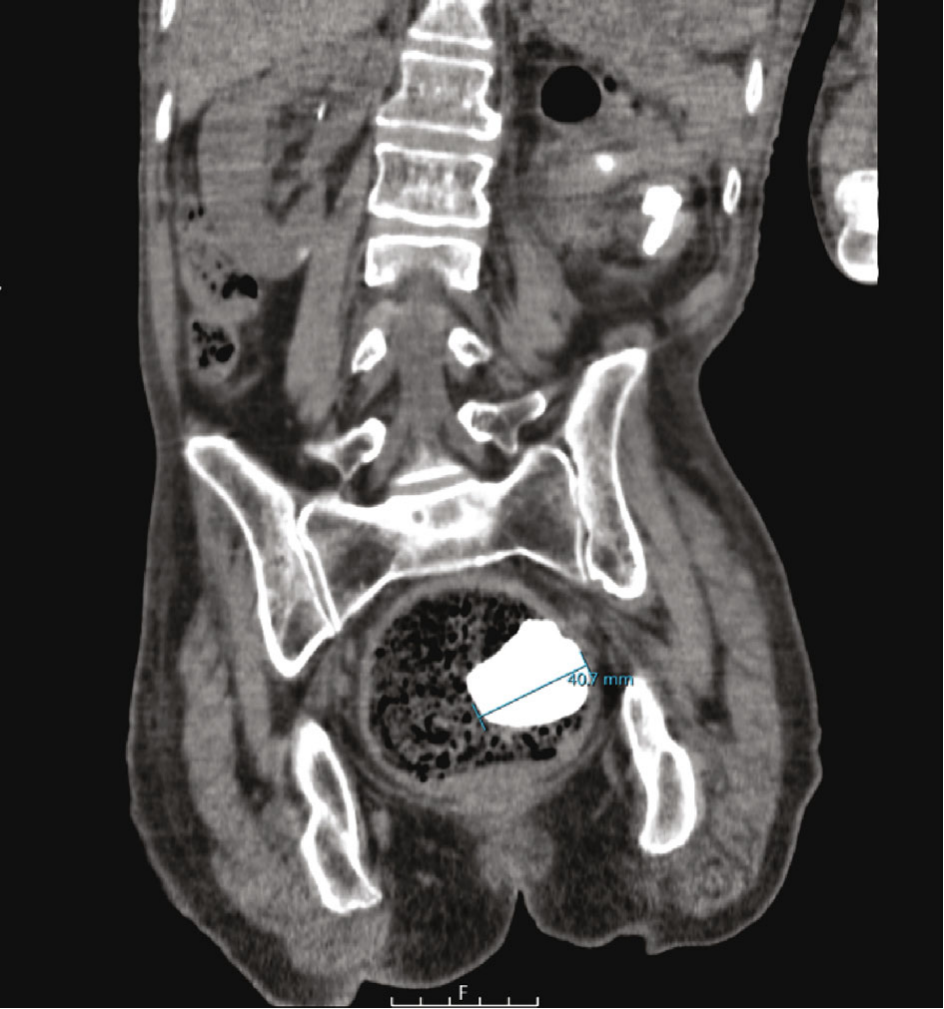
Repeat CT KUB scan showing the staghorn calculus, previously visualized in the fistula, now located inside the rectum.

Due to worsening renal function during hospitalization, repeat imaging (CT KUB) revealed a 4.4-cm staghorn calculus within the rectum (Figures 5 and 6). The CT report highlighted a strong suspicion of a renocolic fistula, facilitating the migration of the stone into the rectum. The stone was subsequently expelled spontaneously through the rectum.

### 2.3. Management and Outcomes

Given her significant comorbidities, the patient's condition was managed conservatively. She was deemed unsuitable for general anesthesia due to the high risk of intubation and was not a candidate for local anesthetic JJ-stent insertion due to her cerebral palsy. Nephrostomy was also ruled out as an option since the in situ staghorn calculus occupied the entire left renal pelvis. Ultimately, after an extended hospital stay, she responded well to conservative treatment with antibiotics. She was given a one-off (STAT) dose of amikacin at presentation and clarithromycin−amoxicillin (1.2 g intravenously three times a day) for 6 days, which was then escalated to ceftriaxone (2 g intravenously once a day) for another 8 days. Her case was also discussed at the Stone Urology Multidisciplinary Team Meeting (MDTM), and it was decided to provide symptomatic treatment only, with no further interventions, as there was a high mortality risk. After improving clinically, she was discharged to her usual place of residence.

### 2.4. Complication: Psoas Abscess

**Figure 7:**
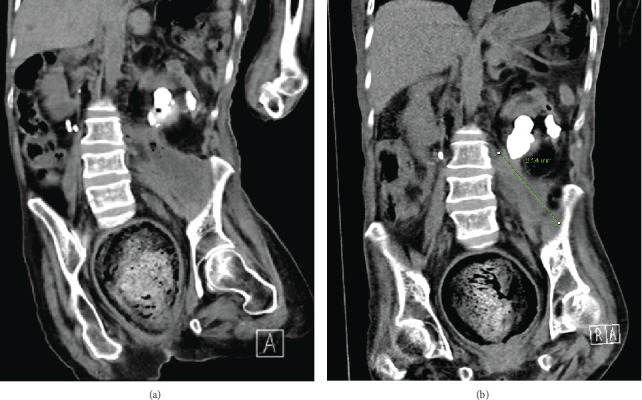
(a, b) CT scan revealing a large left-sided psoas abscess, associated with gas bubbles, observed during the patient's representation several months after initial discharge.

Several months later, she represented to the emergency department with signs of urinary infection (low oral intake, foul-smelling urine, and fevers). A CT KUB scan revealed a large left iliopsoas abscess, measuring 87 × 54 mm, with gas bubbles, alongside bilateral staghorn stones (Figures 7(a) and 7(b)). Conservative management with antibiotics was again recommended due to her extensive comorbidities and high mortality risk. This approach was agreed upon by the Urology Team, the Medical Team, and the Interventional Radiologists. After an extensive course of antibiotics (clarithromycin−amoxicillin 1.2 g three times daily, for 4 weeks), she responded well and was discharged to her usual place of residence. A few weeks later, she attended the emergency department with aspiration pneumonia and sepsis and unfortunately passed away.

## 3. Discussion

This case report presents a rare instance of a staghorn calculus migrating through a renocolic fistula into the rectum. Conservative management for staghorn calculi is generally reserved for patients with significant comorbidities or when their overall survival is expected to be limited, as these individuals are typically not suitable candidates for more invasive procedures [3]. In this case, the decision for conservative management was influenced by the patient's significant comorbidities, including cerebral palsy and active delirium, which heightened the surgical and anesthetic risks. Additionally, her frailty and reduced life expectancy were key factors in opting for a noninvasive approach. As noted in the literature, a conservative approach may also be appropriate for patients with unilateral asymptomatic stones or those who do not experience recurrent UTIs [4].

The management of renocolic fistulas usually involves surgical intervention, as illustrated in Table 1. This includes nephrectomy (partial or total), with or without partial colectomy, and resection of the fistula. However, in our patient, the decision to avoid surgical intervention was made after thorough discussion at the MDTM, where the risks of surgery outweighed the benefits, considering her overall health status.

In this case, the genesis of the renocolic fistula also warrants discussion. Her previous PCNL may have contributed to the formation of the fistula, although this cannot be stated with certainty. Additionally, her recurrent UTIs and the presence of the staghorn calculus could also have played a role as potential causative factors in the development of the renocolic fistula.

Previous case reports have documented instances of staghorn calculi migrating through fistulas (Table 1). However, only one prior case involved a staghorn calculus passing through a renocolic fistula and being found in the large bowel [13], while another case described a staghorn protruding through a renocolic fistula without complete passage [7]. Additionally, two cases reported staghorn calculi found lying just above the psoas muscle [9, 10], and one case involved a staghorn calculus located in the retroperitoneum [8]. There was also a report of a staghorn being expelled through a nephrocutaneous fistula to the external environment [11], and another case where a staghorn migrated through a nephro-ileal fistula, causing small bowel obstruction [12].

Thus, the migration of staghorn calculi beyond the renal pelvis is a rare and surprising radiological finding. Few cases of migrating staghorn stones have been reported, and only one, similar to this case, described the stone being expelled through the rectum. The decision to pursue conservative management in this patient was primarily guided by her comorbidities, distinguishing it from other reported cases where surgical intervention was necessary due to complications such as infection or obstructive uropathy.

## Figures and Tables

**Table 1:** Similar case reports with migrating staghorn calculi.

**Citation**	**Age (years)**	**Type of fistula**	**Findings**	**Management**
Mohsin et al. [8]	48F	Nephrocutaneous fistula	Staghorn calculus was found within the right retroperitoneum abutting the iliopsoas, on the background of previous nephrostomy for an obstructing ureteric calculus. The patient had previously accidentally pulled out nephrostomy tube and had not attended follow-up appointments for years.	The intact stone and the tip of the nephrostomy tube were removed through an elliptical incision that was made over the nephrostomy tract.
Thomas, Deleuze, and Lemaitre [7]	68F	Nephrocolic fistula	Protrusion of a staghorn calculus within the colonic lumen, without complete passage. A retroperitoneal abscess was also present.	Nephrectomy, left segmental colectomy, and splenectomy.
Singh et al. [9]	36F	Ureteric-psoas fistula	Xanthogranulomatous pyelonephritis with calculus migration into the psoas abscess.	Total nephrectomy
Purkait et al. [10]	10F	Nephrocutaneous fistula	The staghorn calculus was embedded in the left psoas muscle. Histology of the kidney showed chronic pyelonephritis.	Subcapsular nephrectomy
Vaidyanathan et al. [11]	29M	Nephrocutaneous fistula	Staghorn calculus was extruded through the fistula to the external environment.	Not mentioned
Kiger, Loehrer, and Sanford [12]	76M	Nephro-ileal fistula	Staghorn calculus causing small bowel obstruction.	Excision of the portion of the bowel containing the staghorn, fistula, and adjacent loop of bowel.
Narins and Segal [13]	56F	Nephrocolic fistula	A large dentritic renal calculus was spontaneously passed per the rectum. Perirenal abscesses were also present.	Conservative management

## Data Availability

No public dataset was used in the creation of this manuscript. Data sharing is not applicable to this article as no new data were created or analyzed in this study.
